# Definitive surgical management for second branchial cleft fistula: a case series

**DOI:** 10.1186/s40463-020-00453-2

**Published:** 2020-08-05

**Authors:** Abhita Reddy, Taher Valika, John Maddalozzo

**Affiliations:** 1 Department of Otolaryngology - Head and Neck Surgery, Northwestern University, 676 N. St. Clair, Suite 1325, Chicago, IL 60611 USA; 2 Division of Otolaryngology – Head and Neck Surgery, Ann & Robert H. Lurie Children’s Hospital, Chicago, IL USA

**Keywords:** Second branchial cleft fistula, Congenital neck mass, Pediatric head and neck surgery

## Abstract

**Background:**

Second branchial cleft fistulae are rare pediatric anomalies managed with surgical excision and, in certain cases, ipsilateral tonsillectomy to prevent postoperative recurrence or wound infection. Limited information is available in the published literature regarding surgical techniques to maximize patient outcomes and minimize recurrence. Our objective was to describe outcomes for the largest series of branchial cleft fistulae excised using a uniform technique based on embryologic principles.

**Methods:**

We conducted a retrospective analysis of pediatric patients who underwent surgery for second branchial cleft fistula using a uniform technique developed by the senior surgeon between 2006 and 2018 at a tertiary care pediatric hospital. The technique involves dissection to the level of the greater cornu of the hyoid bone as the point of transection, which is the landmark for the base of the tonsillar fossa. Data collected included age at surgery, initial presentation, laterality of fistula tract, final pathology, and follow up data. Measured outcomes included fistula recurrence, wound infection, and other complications.

**Results:**

Of 67 patients, 28 (42%) were male and 10 (15%) had bilateral fistulae, for a total of 77 tracts excised. After a median follow up of 31 months, there were no recurrences and one wound infection that was treated successfully with oral antibiotic therapy. No patients underwent tonsillectomy.

**Conclusion:**

Effective management of second branchial cleft fistulae can be challenging. We present the largest cohort of results using a uniform surgical technique performed at a single center that obviates the need for tonsillectomy, and thus represents a less morbid and effective approach with no evidence of recurrence.

## Background

Branchial cleft anomalies are formed due to failure of embryonic structures to obliterate during development. These anomalies are the second most common pediatric congenital head and neck masses, accounting for approximately 20% of cases [1]. Of these, second-cleft lesions comprise 90–95% of all branchial cleft anomalies [2], and can present as true fistulae, sinus tracts, complete isolated cysts, or cartilaginous remnants [3]. Patients with fistulae often present with mucoid drainage from a lateral neck opening that may become infected over time. Given that these patients are at risk for recurrent infection that may lead to abscess formation, definitive management consists of complete surgical excision of the fistula tract.

**Fig. 1:**
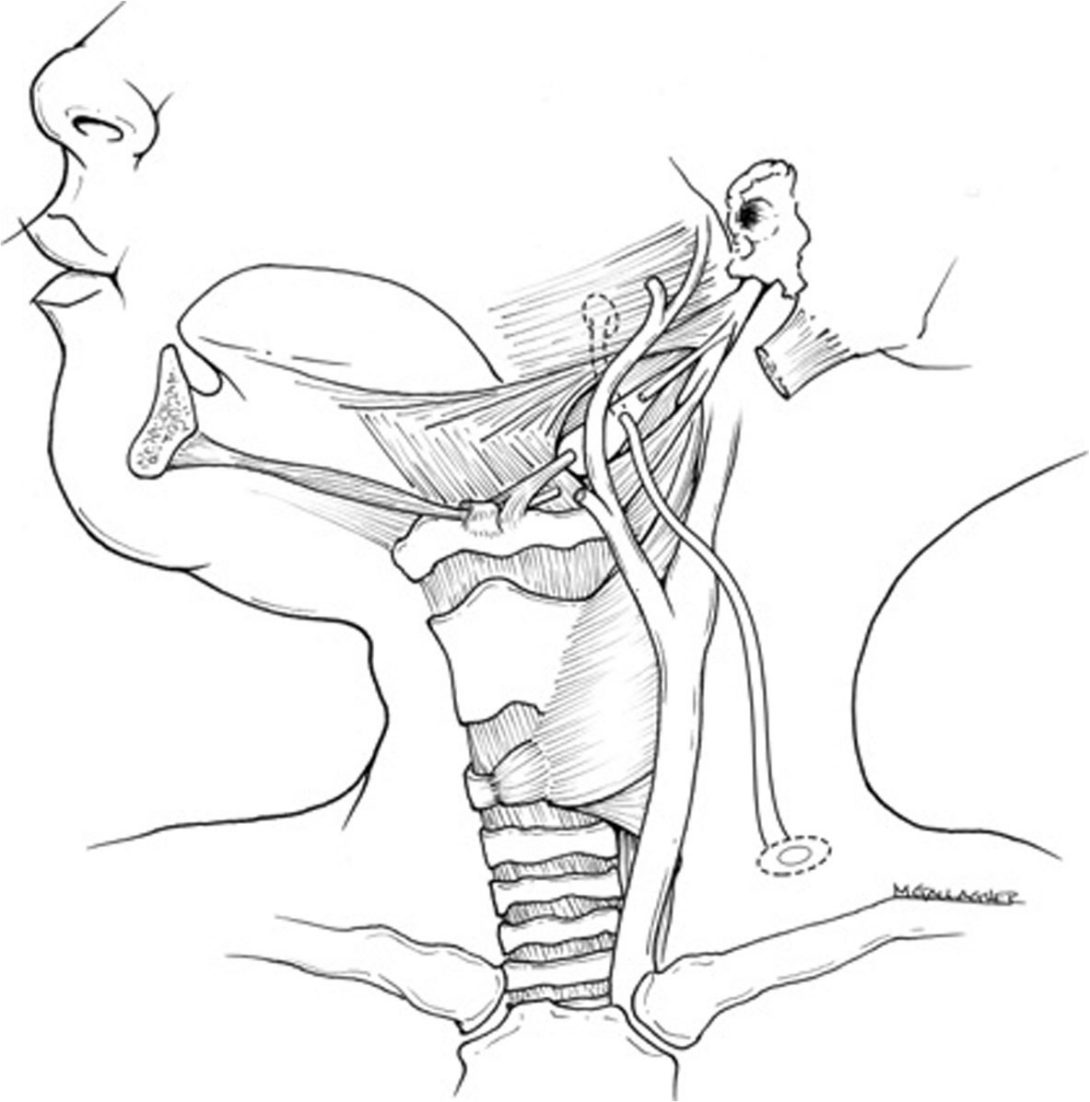
Illustration of left second branchial cleft fistula demonstrating pathway of fistulous tract crossing between internal and external carotid arteries to enter oropharynx just superolateral to greater cornu of hyoid bone

Reported recurrence rates after surgery for second branchial cleft fistulae vary from 3 to 22% [2]. Recurrence has been ascribed to preoperative infection and incomplete excision, though it is likely that recurrence is primarily the result of incomplete excision [4]. Some authors have recommended not only excision of the fistulous tract, but also ipsilateral tonsillectomy given that the tract terminates at the tonsillar fossa, a second branchial pouch derivative (Fig. 1).

We have previously published findings of our experience with 28 patients undergoing surgery for second branchial cleft fistulae, demonstrating no recurrent disease using a surgical technique that employs the hyoid bone as a landmark [5]. In this follow up study, we present 67 cases of second branchial cleft fistulae excision, the largest cohort performed by a single center with a uniform technique reported in the medical literature and compare our findings with those of prior studies. The essential findings in our study reveal the benefits of a uniform technique, utilizing the hyoid bone as a landmark, and thus obviating the need of an ipsilateral tonsillectomy in management of second branchial cleft fistulae, a controversial topic in pediatric otolaryngology.

## Materials and methods

### Study design and patients

We performed a retrospective case series of consecutive patients undergoing surgery for second branchial cleft fistulae, using a uniform technique developed by the senior surgeon at a tertiary care pediatric hospital from 2006 to 2018. Patients were identified from a secure branchial cleft surgical database at our hospital. Patient information was collected at the time of surgery for this database and updated on subsequent visits. Additionally, chart review was performed using the term “branchial cleft” to help identify any missed surgical patients. Consent for this study was approved by the Institutional Review Board at Lurie Children’s hospital and did not require trial registration due to the study’s retrospective nature. Inclusion criteria were patients under 21 years old with previously unoperated unilateral or bilateral second branchial cleft fistulae. Collected data included patient demographics such as sex and age at surgery, date of initial presentation, imaging type and characteristics, syndromic causes of branchial cleft anomalies, laterality of fistula tract, lesion characteristics, any perioperative complications, final pathology report, and date of follow up with any reported complications including wound infection or recurrence. Per our institutional standard, patients did not undergo surgery if they appeared actively infected with at least 1 month between infectious presentation and time of surgery. All patients were given standard postoperative instructions with postoperative antibiotics, usually amoxicillin 40 mg/kg/dose twice daily for approximately 7 days. Patients were seen at least once postoperatively at 1–2 week follow up after surgery, and most patients were also seen 2–3 months postoperatively by an otolaryngologist for clinical exam. Long-term assessment of recurrence and/or complications were assessed by chart review. Additionally, all patient families were phoned at the time of manuscript data collection to confirm chart findings. A total of 101 patient charts were reviewed, and 34 patients with final pathology inconsistent with branchial cleft fistula or with no charted follow up were excluded. Twenty-eight patients from our previous study in 2012 were included in our current data analysis.

### Surgical technique

The same surgical technique was used on all patients throughout the case series. The fistula was canalized with a lacrimal probe, then an elliptical incision was created around the probe. The fistulous tract was identified and skeletonized, then dissected to the muscular division of the middle layer of deep cervical fascia surrounding the strap muscles. The overlying fascia was divided and undermined for improved exposure. The fistulous tract was followed to the hyoid bone, over the hypoglossal nerve and between the carotid bifurcation to the posterior aspect of the hyoid bone where the tract was transected (Fig. 1). This position was posterior, lateral, and superior to the entry point of the hypoglossal nerve to the tongue (Lesser’s triangle), often to the level of tonsillar fossa. The tract was cross-clamped, ligated, and tied with silk suture at or above the level of the hyoid bone. No ipsilateral tonsillectomy was performed on any case.

## Results

Of 67 patients, 28 (42%) were male and 39 (58%) were female. Average age at time of surgery was 4.1 years (4 months-19 years). Prior to surgery, 16 patients had been treated with antibiotics course for presumed branchial anomaly infection. One patient required incision and drainage under ultrasound guidance with interventional radiology for persistent globus sensation and dysphagia. Most patients underwent a computed tomography (CT) scan (27 patients, 40%), some underwent ultrasound (25 patients, 37%), while a few underwent neck magnetic resonance imaging (2 patients, 3%). Imaging was either completed by primary care at the time of initial otolaryngology visit or ordered at the discretion of the senior author (unclear diagnosis, concern for deep cyst, or underlying syndromic association). Eight patients had no imaging, while 5 patients underwent imaging outside the hospital and the images were unavailable at the time of data collection.

Branchial cleft fistulae more commonly affected the right neck (54 patients, 81%), and 10 patients (15%) had bilateral fistulae for total of 77 tracts excised. Six patients were diagnosed with Branchio-oto-renal syndrome on genetic evaluation, 5 of whom presented with bilateral branchial cleft fistulae. The median follow-up time via clinic visit or phone call occurred 31 months after surgery (10 days to 16 years). No patients reported pain or drainage during their postoperative visits. One patient developed a postoperative wound infection that was treated successfully with oral antibiotics as an outpatient. At the time of data collection in 2019, patients’ charts were reviewed for evidence of recurrence by searching primary care and otolaryngology notes. Telephone call follow up was also attempted for each patient’s family to confirm our findings from chart review. There were no reported recurrences or complications in long-term follow up confirmed by phone call in 23 patients and chart review in the remainder of patients.

## Discussion

The purpose of this study was to determine whether a uniform surgical technique for second branchial cleft fistula excision, which uses the hyoid bone as a landmark for definitive surgical management, could avoid the need for ipsilateral tonsillectomy and lower recurrence rates. In this study of a large cohort undergoing this surgical procedure for second branchial cleft fistula, no patients required tonsillectomy and there were no recurrences. Only one patient developed an infection, which was successfully treated with antibiotics.

While branchial cleft anomalies—which may present as a fistula, sinus, cyst or a combination thereof—account for approximately 20% of congenital neck masses, isolated fistulae are rare. Definitive surgical excision for second branchial cleft fistulae is the standard of care. Incomplete excision is considered a major cause of recurrence; thus, some surgeons include ipsilateral tonsillectomy to ensure removal of the fistulous tract. In a study by Kajosaari et al., 46% of second branchial cleft fistulae excision included ipsilateral tonsillectomy, performed by otolaryngologists [2]. Using our excision technique, no tonsillectomies were performed, with no evidence of recurrence in any patient over a median 1-year follow up. Our findings are consistent with those of three prior studies reporting that ipsilateral tonsillectomy does not affect recurrence rates [2, 5, 6]. Ligation at or above the level of hyoid bone is sufficient for complete excision of second branchial cleft fistulae.

**Fig. 2:**
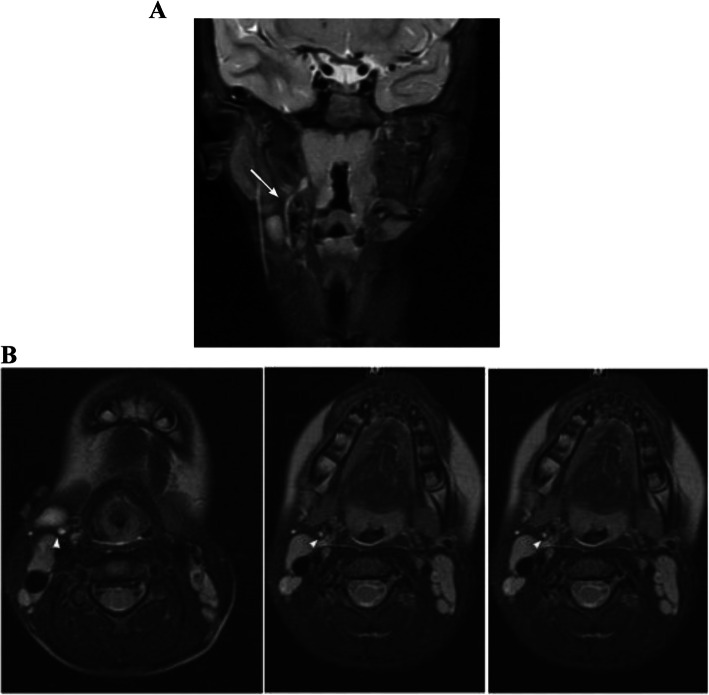
**a** Magnetic resonance coronal T2 imaging demonstrating right second branchial cleft fistulous tract into oropharynx at level of right tonsillar fossa. Arrow pointing to fistulous tract. **b** Progressive magnetic resonance axial T2 imaging demonstrating tract traveling from cyst into insertion at tonsillar fossa. Arrowheads depicting fistulous tract

Based on a consistent embryologic pattern, second branchial cleft fistulae usually pass from an external skin opening at the anterior border of the sternocleidomastoid muscle, between the external and internal carotid arteries, then cross over glossopharyngeal and hypoglossal nerves (Fig. 2a, b). The upper portion of the fistulous tract then ascends superior to the hyoid bone to terminate at the ipsilateral tonsillar fossa.

Using these embryologic and anatomic principles, the senior author developed a uniform technique for second branchial cleft fistulae excision. During development, the second branchial arch forms the lesser cornu and superior body of the hyoid, while the third branchial arch forms the greater cornu and inferior body of the hyoid. Second branchial cleft anomalous tracts pass superficial to the cartilaginous derivative of the second branchial arch (the hyoid) and terminate lateral to the hyoid bone at the second pouch derivative (tonsillar fossa). Thus, using the supero-lateral portion of the hyoid bone, the greater cornu, to identify the base of the tonsillar fossa, complete surgical excision of the second branchial cleft fistulous tract may be achieved.

This is the largest review of second branchial cleft fistulae in medical literature to date. Our data supports findings in current literature that right-sided fistulae are more common [2]. The biggest difference over time in preoperative management for our patients was imaging modality selection. More patients in the updated series underwent ultrasound (22 vs. 3 patients in our previous study) while fewer patients in the updated series underwent CT imaging (5 in our current series vs. 22 in our previous study) [5]. The senior author of this study preferred ultrasound to CT imaging in our updated series cohort given safety from radiation exposure and similar utility in preoperative planning based on senior author’s experience [5].

The limitations of this study included single center results that may not be reproducible. It is possible that patients may have followed-up elsewhere and surgical outcomes may be underreported. We attempted to contact each patient during data collection to confirm lack of recurrence and outside follow up, which is reported in our results. Finally, surgical pathology was completed by different pathologists, which may have led to variation in diagnosing and reporting second branchial cleft fistulae.

Further studies to strengthen current findings could include a prospective randomized control trial studying recurrence rates in a cohort undergoing our surgical technique without tonsillectomy (treatment group) compared to a cohort undergoing surgery with ipsilateral tonsillectomy (control group). Reducing the limitations described previously will help solidify the results of any future publication.

## Conclusions

In the largest cohort of patients with second branchial cleft fistula treated with uniform surgical technique based on embryologic principles at a single center, effective surgical management can be achieved without need for revision surgery or ipsilateral tonsillectomy. This technique thus represents a less morbid and effective approach to the management of second branchial cleft fistula.

## Data Availability

The datasets used and analyzed during the current study are available from the corresponding author on reasonable request.
